# *Pichia cactophila* and *Kluyveromyces lactis* are Highly Efficient Microbial Cell Factories of Natural Amino Acid-Derived Aroma Compounds

**DOI:** 10.3390/molecules23010097

**Published:** 2018-01-02

**Authors:** Ewelina Celińska, Radosław Bonikowski, Wojciech Białas, Anna Dobrowolska, Barbara Słoma, Monika Borkowska, Monika Kubiak, Paulina Korpys, Włodzimierz Grajek

**Affiliations:** 1 Department of Biotechnology and Food Microbiology, Poznan University of Life Sciences, ul. Wojska Polskiego 48, 61-627 Poznań, Poland; wojciech.bialas@up.poznan.pl (W.B.); anna.dobrowolska@up.poznan.pl (A.D.); sloma.barbara@gmail.com (B.S.); monika.borkowska@up.poznan.pl (M.B.); monika.k1404@gmail.com (M.K.); paulinakorpys94@gmail.com (P.K.); wlodzimierz.grajek@up.poznan.pl (W.G.); 2 Institute of General Food Chemistry, Lodz University of Technology, ul. Stefanowskiego 4/10, 90-924 Łódź, Poland; radoslaw.bonikowski@p.lodz.pl

**Keywords:** non-conventional yeast, aroma, bioconversion, 2-phenylethanol, isoamyl alcohol, 2-phenylethyl acetate

## Abstract

The pivotal role of non-conventional yeast (NCY) species in formation of valuable aroma compounds in various food commodities is widely acknowledged. This fact inspires endeavors aiming at exploitation of food-derived NCYs as biocatalysts in natural aromas production. In this study, we isolated, characterized and evaluated aroma-producing capacity of two NCY representatives—*Pichia cactophila* 7.20 and *Klyuveromyces lactis* 6.10 strains. The strains were isolated from food-related habitats—goat-milk regional cheese and Swiss-type ripening cheese, respectively. Aroma profiles generated by the two strains cultured in a general rich medium were analyzed through solvent extraction and GC-MS analysis of the compounds retained in the culture media. Finally, the strains were tested in bioconversion cultures with branched chain- or aromatic amino acids as the sole nitrogen source, to assess capability of the strains towards formation of amino acid-derived aromas. The results showed extraordinary capacity of both strains for production of 2-phenylethanol (at more than 3 g/L) and isoamyl alcohol (approx. 1.5 g/L). A distinctive trait of 2-phenylethyl acetate synthesis at high concentrations (0.64 g/L) was revealed for *P. cactophila* 7.20 strain. Highly valued disulfide dimethyl as well as methionol acetate were identified amongst the aroma compounds synthesized by the strains.

## 1. Introduction

Top five worldwide leading producers of flavors and fragrances, like International Flavours and Fragrances (www.iff.com), Givaudan (www.givaudan.com), Symrise (www.symrise.com), or Firmenich (www.firmenich.com) jointly indicate the urgent need for innovative solutions allowing for production of natural aroma compounds. Traditionally, highly-valued aroma compounds have been acquired from plant and animal resources. However, high market demand for the aromas has led to gradual exhaustion of some of these resources, especially of animal origin. This in turn prompted enforced legislatives banning their further exploitation and, in many cases, made the products unavailable on the legal market. Moreover, plant-derived natural resources are highly susceptible to seasonal variations, and/or hard-to-foresee climate-geographical factors, which significantly perturb the production processes.

Another important route of aroma compounds provision to the global market is chemical synthesis. Currently operating chemical synthesis processes comprise complex reactions, requiring highly specialized and costly installations, applying extreme physical conditions, as well as toxic solvents and catalysts, altogether contributing to substantial burden imposed on the natural environment. Importantly, chemical synthesis processes are currently the most efficient and the most economically-feasible processes of the aroma compounds synthesis, which is mirrored by the low market price and high accessibility of such products. Nevertheless, it is not always possible to obtain a synthetic equivalent of a chemical compound with nature-identical properties desired by the consumer. Furthermore, lack of the consumer acceptation significantly limits the global demand for synthetic aroma compounds. In contrast to synthetic aroma compounds, nature-derived aromas are commonly accepted supplements in food, cosmetics and high-grade household chemistry. For example, most of rose-like aroma compound, 2-phenylethanol (2-PE), is chemically synthesized *via* an efficient chemical synthesis process from benzene. However, exploitation of such a primary substrate makes the product useless in flavoring of cosmetics or foodstuff. The use of chemically synthesized 2-PE as a flavoring agent is restricted in the United States and Europe [1]. 

For all the mentioned above reasons, future strategies or already implemented innovative solutions by the major-players on the global market of aroma compounds are strongly embedded in biotechnological processes, which constitute the response to the current market limitations. According to European regulation on flavors (EEC No. 1334/2008), aroma chemical compounds can be labeled as “natural” when ‘the flavoring substance is obtained by appropriate physical, enzymatic or microbiological processes from material of vegetable, animal or microbiological origin either in the raw state or after processing’, which opens the way for biotechnological production of natural aroma compounds. This in turn makes biotechnology a powerful alternative in aroma compounds production sector.

Amongst microbial species potentially assisting aroma compounds production, yeasts occupy an important place [2]. The leading role within this area has been usually assigned to food-habitat-related *Saccharomyces cerevisiae*, a species having pivotal role in foodstuff production, contributing importantly to organoleptic properties of the foods [3]. However, along with the growing amount of knowledge accumulated on this subject, it became obvious that the other yeast species play an equally important role in development of unique aromas of the food commodities [2,4]. The definition of the so-called “non-conventional” yeast (NCY) species, differs between individual reports on this subject [5,6], but most agree to assign this term to non-*S. cerevisiae* strains. This growing awareness of the pivotal role of NCYs in development of unique and desired aroma profiles of foods and beverages has inspired endeavors aiming at isolation and screening of the yeasts for their aroma-producing potential [4,7,8,9]. The food-related environments are the preferred source of microbial isolates, as they usually ensure isolation of safe microbiota. This in turn enables their application in biotechnological processes without safety concerns. Among these, an important role is assigned to *Kluyveromyces marxianus*, exhibiting great capacity in production of acetate esters, being the key odorants of numerous foods and beverages [7,10,11], and 2-phenylethanol, a valuable aroma compound of rose-like odor [12,13].

Although the microbial potential for de novo aromas formation is pronounced, the concentrations of the targeted compounds originating from the central carbon metabolism are usually very low [14]. On the other hand, synthesis of the aroma compounds in a course of biocatalytic conversion of a structurally-related precursor into the targeted chemical molecule frequently appears to be a superior strategy, which allows for accumulation of a desired flavor compound in significantly higher amounts [2]. Examples of such bioconversions have been extensively described in the literature, for instance, synthesis of vanillin from ferulic acid or eugenol, γ-decalactone—from ricinoleic acid, isoamyl alcohol—from l-Leu, and 2-phenylethanol—from l-Phe [14,15]. Microbial-derived aroma compounds mainly origin from two metabolic meta-pathways involving lipids and amino acids conversions; or, in substantial proportion, result from lipases/esterases action. Amino acid-derived aromas are generated in an extensive amino acid turnover net. The metabolic pathway of branched chain amino acids (l-Val, l-Leu, l-Ileu), aromatic amino acids (l-Phe, l-Tyr, l-Trp) and l-Met catabolism has been initially described by Ehrlich [16], and investigated in great scrutiny in the following studies (e.g., [3,17]).

In the present study we report on isolation and characterization of new NCY representatives originating from foodstuff habitats and exploration of their aroma-producing capacity in a series of batch cultivations. Initial characterization of the isolates by the cell and colony morphology was followed by identification of the strains by rDNA region sequencing, and description of aroma profiles generated by the strains through GC-MS analysis of the compounds retained in the culture medium. Finally, assessment of the NCY strains potential in bioconversion of amino-acid precursors into valuable aroma compounds was conducted.

## 2. Results and Discussion

### 2.1. Isolation of the Yeast Monocultures and Taxonomic Classification

According to the literature data, amongst the most rich sources abundant in NCY microbiota, three are of major importance, namely: Dairy products, in particular cheeses and kefyr (e.g., [18,19]), meat (e.g., [20]), and fruits (e.g., [4]). Individual properties of these foodstuffs predispose different yeast species to populate those environmental niches. In this study, we focused on dairy products to be used as the microbiota sources, recruiting traditional homemade goat-milk regional cheese manufactured with autochthonic microbiota. Dairy products’ microbiota is manly dominated by lactic acid bacteria, accompanied by occurrence of *Enterobacteriaceae* (and some other *Proteobacteria*), *Actinobacteria*, *Firmicutes* and *Bacteroidetes*. Yeast species usually occur as spontaneous microbiota, not provided in a starter, but their presence is of high importance in dairy products for their pH-modulating role, as well as proteo- and lipolytic activities [18,19]. In some specific cases, yeast strains belonging to *K. lactis, K. marxianus*, and *D. hansenii* species are contained in the starter cultures used in industrial production of cheeses [18]. It has been demonstrated in a number of scientific reports that traditional food commodities, manufactured with indigenous, spontaneous microbiota (like the goat-milk regional cheese in this study) constitute a rich source of unique microbial strains (e.g., [21]). According to the literature data, amongst the NCY species most frequently isolated from various cheeses one can identify primarily different *Candida* species, followed by *K. marxianus* and *K. lactis, Pichia* spp. *Rhodotorula* spp., *Trichosporon* spp., but also *Wickerhamomyces*, *D. hansenii*, *K. fragilis*, *K. bulgaricus*, *Geotrichum candidum*, *Torulopsis sphaerica*, and *Torulospora delbrueckii*, which are found both on the surface and in the core of the cheeses [18,19,21]. A detailed analysis of microbiota of “Rokpol” cheese (manufactured in Southern Poland) showed prevalence of yeast occurrence on the surface compared to the cheese interior, and domination of *Candida* spp. (*C. famata*, *C. spherica*, *C. intermedia*), followed by *Geotrichum*, with occasional occurrence of *Y. lipolytica* [9].

In the current study, the primary pre-propagation of the samples’ microbiota was done to promote growth of fungi and diminish bacterial counts due to decreased pH, according to the guidelines by Kurtzman et al. [22]. Fungi growing on YGC plates were subsequently differentiated into yeast or molds based on the macroscopic and microscopic morphology. Yeast and mold counts in Swiss-type and goat-milk cheese pre-propagated samples equaled to 10^7.23 and 10^5.3 cfu/g for the former, and 10^10.87 and 10^9 cfu/g for the latter, respectively. Selected strains having yeast-like colony morphology were further analyzed through microscopic observations, and screening for “non-conventional” cell morphology, based on the guidelines provided by Kurtzman et al. [22]. Subsequently, the selected strains were subjected to sequencing of rDNA fragment of genomic DNA for taxonomic identification. The highest proportion of the isolates was classified to *Candida citrii* (46%) species, followed by *Pichia fermentans* (30%). A single strain (7.20) isolated from the goat-milk cheese was classified in *Pichia cactophila* species. *P. cactophila* species, naturally occurs in cactuses exudates, but was also frequently isolated from fermented food commodities, like Italian traditional cheeses (e.g., buffalo mozzarella, a typical cheese from Southern Italy) [22]. The only representative of *K. lactis* (strain 6.10) species in this study was isolated from the Swiss-type ripening cheese. *K. lactis* strains are typically associated with fermented dairy products [22], which stays in full agreement with our result. The two rare isolates were subjected to further studies.

### 2.2. GC-MS Analysis of Aroma Compounds Produced by the Selected NCY Strains

**Figure 1:**
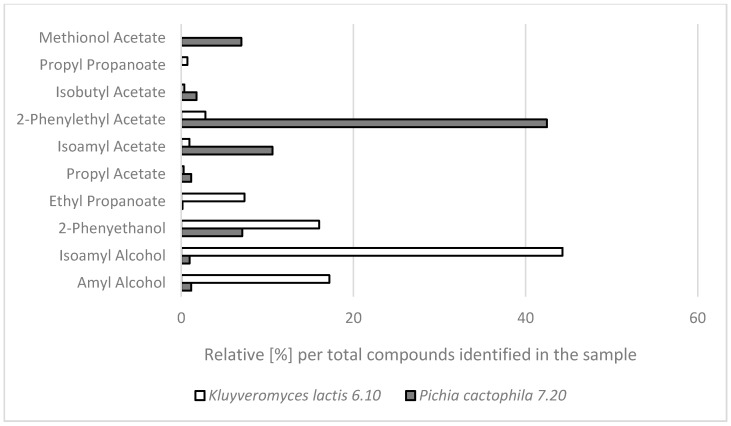
Aroma compounds profiles generated by the two selected NCY isolates—*P. cactophila* 7.20 and *K. lactis* 6.10. Aroma compounds retained in the culture medium were first extracted with dichloromethane and analyzed through GC-MS. The values presented in the figures are mean percentages, and represent relative abundance of an indicated compound per total amount of identified compounds present in the sample. Empty bars: *K. lactis* 6.10; grey bars: *P. cactophila* 7.20. Each sample was analyzed in duplicate.

To get an insight into the landscape of aroma compounds synthesized by the selected NCY strains, a GC-MS analysis of the culture medium supernatants was conducted. Most of the reports on screening of microorganisms for their aroma-producing potential exploits headspace-solid phase microextraction (HS-SPME) followed by GC-MS analysis as the method of choice for its high-throughput character. In this study we applied solvent extraction of the aroma compounds from the cultures, to define the spectrum of the compounds retained in the media, but primarily, to determine their actual concentrations. The strains were cultured in a complete medium, rich in potential precursors for natural aroma compounds. The results of GC-MS analysis are shown in Figure 1. Aroma profile generated by *P. cactophila* 7.20 strain was represented by 2-phenylethyl acetate, followed by isoamyl acetate (IUPAC: 3-methyl-1-butyl acetate), 2-phenylethanol accompanied with methionol acetate (IUPAC name: 3-methylsulfanylpropyl acetate), amyl alcohol (IUPAC: 2-methyl-1-butanol), isoamyl alcohol (IUPAC: 3-methyl-1-butanol), ethyl acetate (ethyl etanoate), ethyl propanoate, propyl acetate and isobutyl acetate (2-methylpropyl acetate). While the aroma profile generated by *K. lactis* 6.10 was generally highly corresponding to the one produced by *P. cactophila*, no methionol acetate was identified in the former. Moreover, propyl propanoate which was absent from *P. cactophila*’s profile—was identified in the *K. lactis* culture medium.

### 2.3. Bioconversion Cultures

**Figure 2:**
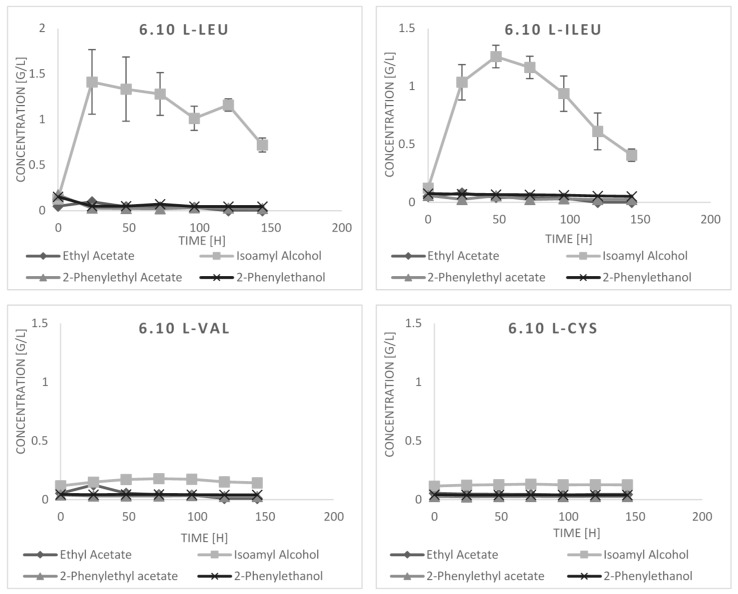

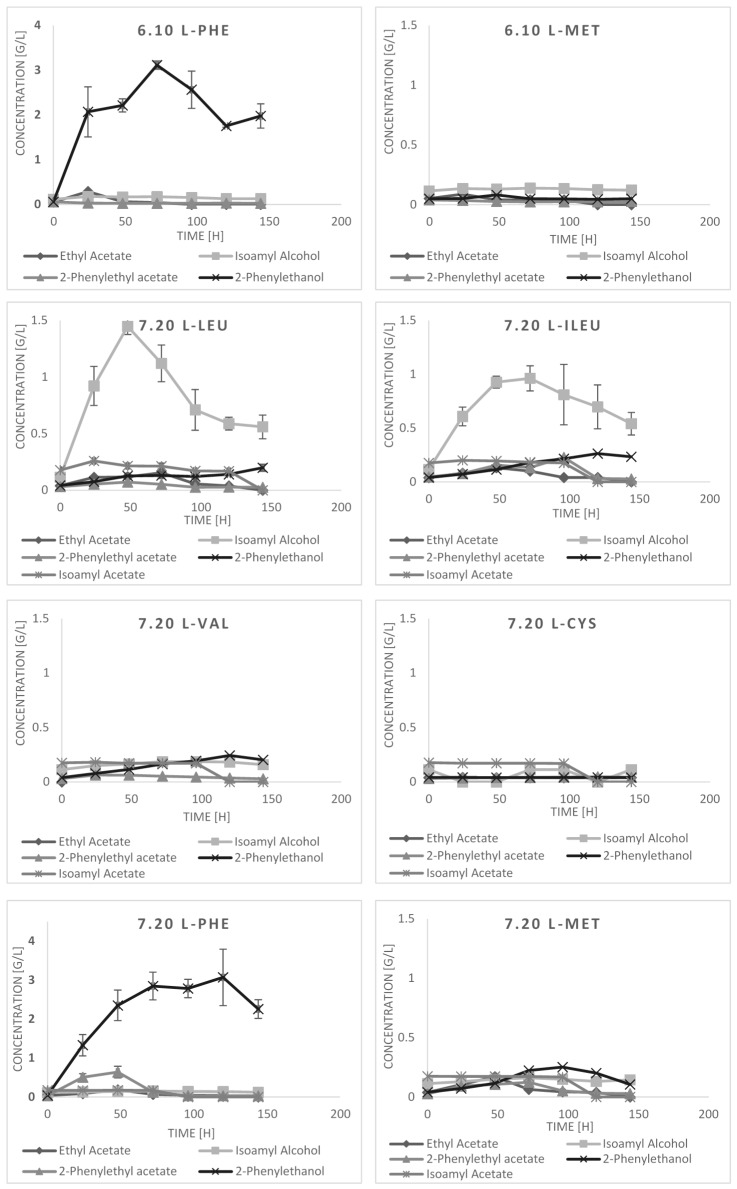
Production of aroma compounds by *P. cactophila* 7.20 and *K. lactis* 6.10 in the bioconversion cultures. The figures represent kinetic analysis of aroma compounds production in a course of bioconversion of an individual amino acid precursor into the respective aroma compounds. The title of each figure refers to the cultured strain (6.10 or 7.20) and amino acid precursor contained in the medium (l-Leu, l-Ileu, l-Val, l-Cys, l-Phe, l-Met). The results show concentration of aroma compounds retained in the culturing media, analyzed through solvent extraction and GC analysis. Y axis: concentration of aroma compounds in g/L (please note different maximum values on the Y axis in the individual schemes). X axis: time of culturing in h. Each culture was conducted in three independent runs (biological triplicate) and each sample withdrawn from the cultures was analyzed in duplicate (technical duplicate). Error bars represent ± SD from the replicates.

Amino acid degradation pathway and lipase/esterase action are metabolic activities of crucial importance for generation of aroma compounds desired in a number of foodstuffs, chemical industry and perfumery [3,14,17,23,24]. Therefore, in this study we set a series of bioconversion cultures, oriented towards production of amino acid-derived aroma compounds. The metabolites buildup was analyzed based on their concentration retained in the culture medium, to comply with the standard methods of biotechnological production of any chemical compound. Strains *K. lactis* 6.10 and *P. cactophila* 7.20 were grown in a production medium—YNB- and glucose-containing medium, supplemented with individual amino acids as the sole nitrogen source. The results of the production cultures are shown in Figure 2. Upon supplementation with l-Leu, the major buildup of its derivative—isoamyl alcohol, was observed within the first 24–48 h, reaching a peak value of 1.41 ± 0.35 g/L ± SD at 24 h or 1.45 ± 0.07 g/L ± SD at 48 h for 6.10 and 7.20 strain, respectively. Likewise, external addition of l-Ileu triggered buildup of isoamyl alcohol up to 1.26 ± 0.09 or 0.93 ± 0.05 g/L ± SD at 48 h of culturing for 6.10 and 7.20 strain, respectively. Supplementation of the cultures with l-Phe greatly enhanced accumulation of 2-phenylethanol in the culture medium, up to 3.11 ± 009 g/L ± SD at 72 h in the 6.10 strain culture, and up to 2.85 ± 0.35 g/L ± SD in the 7.20 strain culture at the corresponding time. Strikingly, supplementation with l-Phe promoted synthesis of valuable 2-phenylethyl acetate only in *P. cactophila* 7.20 strain cultures, reaching 0.64 ± 0.15 g/L ± SD at 48 h of culturing. This compound was not identified in the *K. lactis* 6.10 bioconversion cultures at detectable amounts. Due to highly valued aroma properties of 2-phenylethyl acetate, and the observed high capacity of *P. cactophila* 7.20 strains towards its production, this ester could constitute the target aroma compound of the 7.20 strain’s cultures preceded by optimization procedures.

Similar studies assessing aroma-producing capacity of yeasts were conducted recently [25]. In that study, synthesis of four higher alcohols and their corresponding acetate esters was analyzed in *S. kudriavzevii, S. uvarum,* and *S. cerevisiae* cultures under supplementation with a precursory amino acid. According to their results, *S. uvarum* exhibited relatively high potential in the esters formation (2-phenylethyl acetate, ~1.25 mg/L; isoamyl acetate, ~0.6 mg/L). On the other hand, *S. kudriavzevii* showed better capacity for 2-phenyethanol synthesis (~250 mg/L). Amyl and isoamyl alcohols, under supplementation with a corresponding precursor, were synthesized at comparative levels by the three strains under study (~100 mg/L and ~175 mg/L, for each compound respectively). Obviously direct comparison of the results obtained in that and our study is impossible due to differences in the experimental and analytical procedures. We have applied less throughput method of solvent extraction followed by GC analysis of the compounds retained in the medium, while in the aforementioned paper HS-SPME-GC was exploited for assessment of the compounds concentration in the headspace of the cultures.

Both of the strains analyzed in this study produced competitive amounts of 2-PE, in comparison to the literature data (without in situ product removal techniques; ISPR). 2-PE is a valuable aroma compound of great scientific and industrial interest [26]. Several reports have previously demonstrated high capability of various yeast species in production of this aroma compound in a course of l-Phe bioconversion, e.g., *S. cerevisiae* mutant strain producing 3.52 g/L, starting from 1.39 g/L prior to the process conditions optimization [27], industrial *S. cerevisiae* strain producing 2.1 g/L, starting from 1.7 g/L prior to optimization [8], *K. marxianus* wild type strain producing initially up to 0.9 g/L [7], and 5.6 g/L after culture parameters optimization [12], *P. fermentans* (0.5 g/L; [28]), *Aspergillus niger* (1.9 g/L; [29]), or *Zygosaccharomyces* (0.8 g/L; [7]). In our previous study, we have reported that *Y. lipolytica* strain is able to produce relatively high amounts of 2-phenyethanol, reaching up to 2 g/L, without medium optimization [30,31]. In *P. cactophila* 7.20 production cultures, 2-phenylethanol and its acetate ester were intensively synthetized during the logarithmic growth phase of the cultures, which was then followed by a drop in the ester formation accompanied by its consumption and further buildup of the alcohol. The peak concentrations of 2-phenylethanol in the l-Phe-supplemented cultures (without ISPR techniques and culture optimization) were of 3.11 ± 0.09 g/L ± SD at 72 h for 6.10 strain, and of 3.07 ± 0.725 g/L ± SD at 120 h for 7.20 strain. Although a direct comparison of here presented results and literature data is not completely correct due to obvious experimental differences, it can be stated that both here obtained wild type strains show great potential towards production of 2-PE, which constitutes a promising starting point for future optimization studies. As demonstrated by the former reports mentioned above, optimization of culturing parameters may significantly improve the bioconversion processes performance.

The bioconversion cultures of *K. lactis* 6.10 and *P. cactophila* 7.20 strains were accompanied by an additional run of GC-MS analyses, to determine if any new aroma compounds appeared in the culturing media, apart from those pre-selected after the primary cultivation run. The analysis revealed no new aroma compounds being synthesized in the amino acid-supplemented minimal medium, apart from disulfide dimethyl, an aroma compound of sulfurous, vegetable, cabbage and onion flavour, particularly desired in meat and cheese-containing foods. Its production was triggered by supplementation with l-Met in both strains’ cultures. Surprisingly, synthesis of methionyl acetate (3-methylsulfanylpropyl acetate), identified in the *P. cactophila* 7.20 strains cultures by primary GC-MS analysis, remained at the marginal level under l-Met supplementation. As reported by Etschmann et al. [32], high l-Met supplementation (>2 g/L), as applied in the current study, promoted synthesis of another sulfur compounds, namely methionol and its acetate ester in *S. cerevisiae* cultures. High efficiency of l-Met conversion into methionol and its aldehyde was also recently reported by Seow et al. [33] for *S. cerevisiae* and *K. lactis*. On the other hand, production of disulfide dimethyl under l-Met provision in the culture media was not reported in those studies. Supplementation with the other sulfuric amino acid, l-Cys, did not promote synthesis of this compound.

## 3. Material and Methods

### 3.1. Environmental Samples and Monocultures Isolation

Swiss-type ripening cheese and goat-milk regional ripening cheese from Eastern Poland region were the sources of microbiota isolation. The samples (10 g) were suspended in 90 mL of PreM medium (g/L: NH_4_H_2_PO_4_, 5; KH_2_PO_4_, 2.5; MgSO_4_ × 7H_2_O, 1; glucose, 10; trace elements solution MII [34], 1 mL; pH 5.2) and homogenized in a stomacher homogenizer. The suspension was transferred into Erlenmayer flasks and incubated at 30 °C, 200 rpm, for 24 h. The pre-cultures were serially diluted in peptone water and plated on YGC medium (g/L: glucose, 20; yeast extract, 5; chloramphenicol, 0.1; agar, 15; pH 6.6). After 48 h at 30 °C, colonies were counted, and non-mold colonies were re-streaked onto YPD plates (g/L: yeast extract, 10; glucose, 10; peptone, 20; agar, 15) for monocultures selection. Selection was based on macroscopic observation of the colony growth character (global morphology), as well as on the microscopic image of the cells. Microscopic observations of the yeast morphology was carried out using Zeiss, Axiovert 200 Inverted Microscope and corresponding software (Carl Zeiss Microscopy, LLC, New York, NY, USA). The microscopic preparations were fixed, stained with crystal violet for better visualization of the cell’s structural details and observed under 1000× magnification, with immersion oil. The preferred cells were ovoidal, obovoidal, bacilli-form, elongate shape, or spherical/subglobose, but with substantial differences in size in comparison to conventional *S. cerevisiae*-type cells (according to Kurtzman and Fell, 1998). Strains meeting these criteria were deposited as glycerol stocks at −80 °C, in own collection of Department of Biotechnology and Food Microbiology, Poznan University of Life Sciences.

### 3.2. Identification of the Isolates—rDNA Region Sequencing

Identification of the isolates was conducted through sequencing and blasting against GenBank database of a genomic DNA region covering 5.8S rDNA gene, a fragment of 18S rDNA gene, and the two ITS regions, altogether of ~2 kbp [35]. Prior to amplification, the cells were disrupted by 10 min incubation at 95 °C, in STET buffer (1% Triton X-100, 20 mM Tris-HCl, 2 mM EDTA, pH 8.0), followed by 10 min centrifugation at 4 °C, 22,639× *g* (Biofuge R, Hareus, Hanau, Germany). The following PCR conditions were applied: primers: NS3: GCAAGTCTGGTGCCAGCAGCC, ITS4: CTCCGCTTATTGATATGC; *Pwo* DNA polymerase WALK (A&A Biotechnology, Gdynia, Poland), the reaction mixtures were prepared according to the manufacturer’s recommendation; temperature profile: 94 °C for 5 min, (94 °C for 30 s, 55 °C for 1 min, 72 °C for 2 min) × 25, 72 °C for 5 min, 4 °C hold. Amplicons were verified through 1% agarose gel electrophoresis in TBE buffer. Provided the correct length of the amplicons and a single band occurrence, the remaining volume of PCRs was purified using Clean-Up kit (A&A biotechnology, Gdynia, Poland), and the purified fragments were sequenced (Genomed sequencing facility, Warsaw, Poland), blasted against GenBank Nucleotide database using BLASTn tool [36].

### 3.3. GC-MS Analysis

The yeast biomass was transferred from YPD agar into Erlemayer flasks containing 50 mL of liquid YPD medium (g/L: yeast extract, 10; glucose, 10; peptone, 20) and cultured at 30 °C, 150 rpm, for 48 h. After centrifugation at 2037× *g*, for 10 min (Biofuge R, Hareus, Hanau, Germany), and the supernatants were passed through a syringe filter (0.45 µm, Millex, Millipore, Burlington, MA, USA). For aroma compounds extraction, dichloromethane was added to the supernatants at 1.3%, mixed intensively, and left for phases separation. The organic phase (1 µL) was analyzed using Pegasus 4D GCxGC-TOFMS apparatus (LECO) equipped with a BPX-5 capillary column. The temperature programme was 50 to 300 °C at 6°/min, carrier gas: helium, flow 1 mL/min. Mass spectra were collected using a time-of-flight mass spectrometer, the settings were as follows: ion source temperature 200 °C, ionization energy 70 eV, acquisition voltage 1550 V, scan range 33–600 at 30 spectra/s. Data were analyzed using LECO ChromaTOF software (v. 4.50.8.0). The results were presented as a list of compounds contained in the samples.

### 3.4. Bioconversion Cultures

Cultivations were conducted in Erlenmayer shake-flasks (100 mL total volume, 25 mL culture volume) in the following medium formulation (g/L): YNB *w*/*o* AA and ammonium sulfate, 0.5, K_2_HPO_4_, 10, glucose, 40, trace elements solution MII [34], 1 mL, MgSO_4_ × 7H_2_O, 0.5. The medium was supplemented with amino acids: l-Leucine (l-Leu), l-Valine (l-Val), l-Isoleucine (l-Ileu), l-Cysteine (l-Cys), l-Methionine (l-Met), l-Phenylalanine (l-Phe), to the final concentration of 50 mM. Amino acids stock solutions were prepared in H_2_O, and sterilized through filtration (0.22 µm, Millex, Millipore). Samples were collected in 24 h intervals and stored at −20 °C until analyzed through GC. All batch cultivations were conducted in three independent runs (biological triplicate) and each sample withdrawn from the cultures was analyzed in technical duplicate. Thus, the resultant values constitute a mean value of six readouts at each time point with a corresponding standard deviation ± SD.

### 3.5. Gas Chromatography

After thawing, the samples were centrifuged (15 min, 22,639× *g*; Biofuge R, Hareus), and the supernatants were passed through 0.45 µm filter (Millex, Millipore). Extraction of the aroma compounds was conducted through mixing equal quantities of dichloromethane, vigorous shaking, and phases separation via centrifugation (15 min, 22,639× *g*; Biofuge R, Hareus). The GC analysis of the organic phase (1 µL) was conducted using Agilent Technologies (Santa Clara, CA, USA) 7890A System gas chromatograph equipped with a Zebron™ ZB-WAXplus™ GC capillary column and FID detector. Mobile phase: Hydrogen (30 mL/min), air (300 mL/min), helium (20 mL/min). The oven programme was as follows: 80 °C for 1 min, 80 °C to 120 °C, at 20 °C/min, followed by 120 °C to 220 °C, at 120 °C/min, and final hold at 220 °C for 3 min. Temperature of detector –250°C. Data were analyzed using Agilent Chemstation software. Compounds were identified and quantified through comparison with analytical standards of known concentrations. Analytical standards were purchased from Sigma-Aldrich (St. Louis, MO, USA).
